# Fixation of genetic variation and optimization of gene expression: The speed of evolution in isolated lizard populations undergoing Reverse Island Syndrome

**DOI:** 10.1371/journal.pone.0224607

**Published:** 2019-11-11

**Authors:** Maria Buglione, Simona Petrelli, Valeria Maselli, Martina Trapanese, Marco Salvemini, Serena Aceto, Anna Di Cosmo, Domenico Fulgione

**Affiliations:** Department of Biology, University of Naples Federico II Naples, Naples, Italy; Tierarztliche Hochschule Hannover, GERMANY

## Abstract

The ecological theory of island biogeography suggests that mainland populations should be more genetically divergent from those on large and distant islands rather than from those on small and close islets. Some island populations do not evolve in a linear way, but the process of divergence occurs more rapidly because they undergo a series of phenotypic changes, jointly known as the Island Syndrome. A special case is Reversed Island Syndrome (RIS), in which populations show drastic phenotypic changes both in body shape, skin colouration, age of sexual maturity, aggressiveness, and food intake rates. The populations showing the RIS were observed on islets nearby mainland and recently raised, and for this they are useful models to study the occurrence of rapid evolutionary change. We investigated the timing and mode of evolution of lizard populations adapted through selection on small islets. For our analyses, we used an *ad hoc* model system of three populations: wild-type lizards from the mainland and insular lizards from a big island (Capri, Italy), both *Podarcis siculus siculus* not affected by the syndrome, and a lizard population from islet (Scopolo) undergoing the RIS (called *P*. *s*. *coerulea* because of their melanism). The split time of the big (Capri) and small (Scopolo) islands was determined using geological events, like sea-level rises. To infer molecular evolution, we compared five complete mitochondrial genomes for each population to reconstruct the phylogeography and estimate the divergence time between island and mainland lizards. We found a lower mitochondrial mutation rate in Scopolo lizards despite the phenotypic changes achieved in approximately 8,000 years. Furthermore, transcriptome analyses showed significant differential gene expression between islet and mainland lizard populations, suggesting the key role of plasticity in these unpredictable environments.

## Introduction

### Island evolution and syndromes

Island populations have provided insights on evolutionary processes such as species radiations [1] and the generation of evolutionary novelties [2]. Island endemic species are typically differentiated from their mainland progenitors in behavior, morphology, and genetics, often resulting from long-term evolutionary change [3]. Genetic variation in insular population is due to the drift effect, selection, the rise of novel mutations and new alleles from secondary immigrations [4].

The ecological theory of island biogeography has been extended to an evolutionary scale, suggesting that populations on large and distant islands should be more genetically divergent from the mainland than populations on small and close islands [5]. However, in some cases, it is possible to observe extraordinary changes in lizards on islands close to the mainland, as variation of head morphology or a wide range of skin colour, from yellow to dark blue [6–10]. Some island populations might not evolve with a fixed genomic mutational rate since they undergo Island Syndrome, which rapidly drives their divergence [11–16]. The notion of accelerated evolution in island populations has been disputed [17,18], hence their rate of evolution remains an open question.

While the mainland populations evolve more slowly than their island relatives, small island populations evolve very quickly over brief periods, ranging from decades to thousands of years. The rate of evolution for island species slows down after the initial period of accelerated change, approaching rates observed on the mainland [19,20]. Isolation time plays a significant role in the rate of evolution: for example, body size variation occurs at a faster pace during the initial period of isolation [21].

The changes related to the insular syndrome may be a direct effect of gene expression more than mutational events that affect the speed of phenotypic evolution [22]. A special case is the Reversed Island Syndrome (RIS), a suite of shifted traits, including higher food intake rates, increased aggressiveness, strong sexual dimorphism and more resource allocation into reproduction compared to mainland relatives. RIS drives adaptation in the populations living in an unpredictable environment, such as small islets, even close to the mainland (as described in [22–25]).

### Aim, species model and tools

According to the RIS hypothesis, the rates of genetic changes in isolated populations should be less than rates observed in a population undergoing the syndrome. To verify this hypothesis, an *ad hoc* model system, including two isolated monophyletic populations, one under RIS and the other not affected by the syndrome, may be an interesting case study.

**Fig 1:**
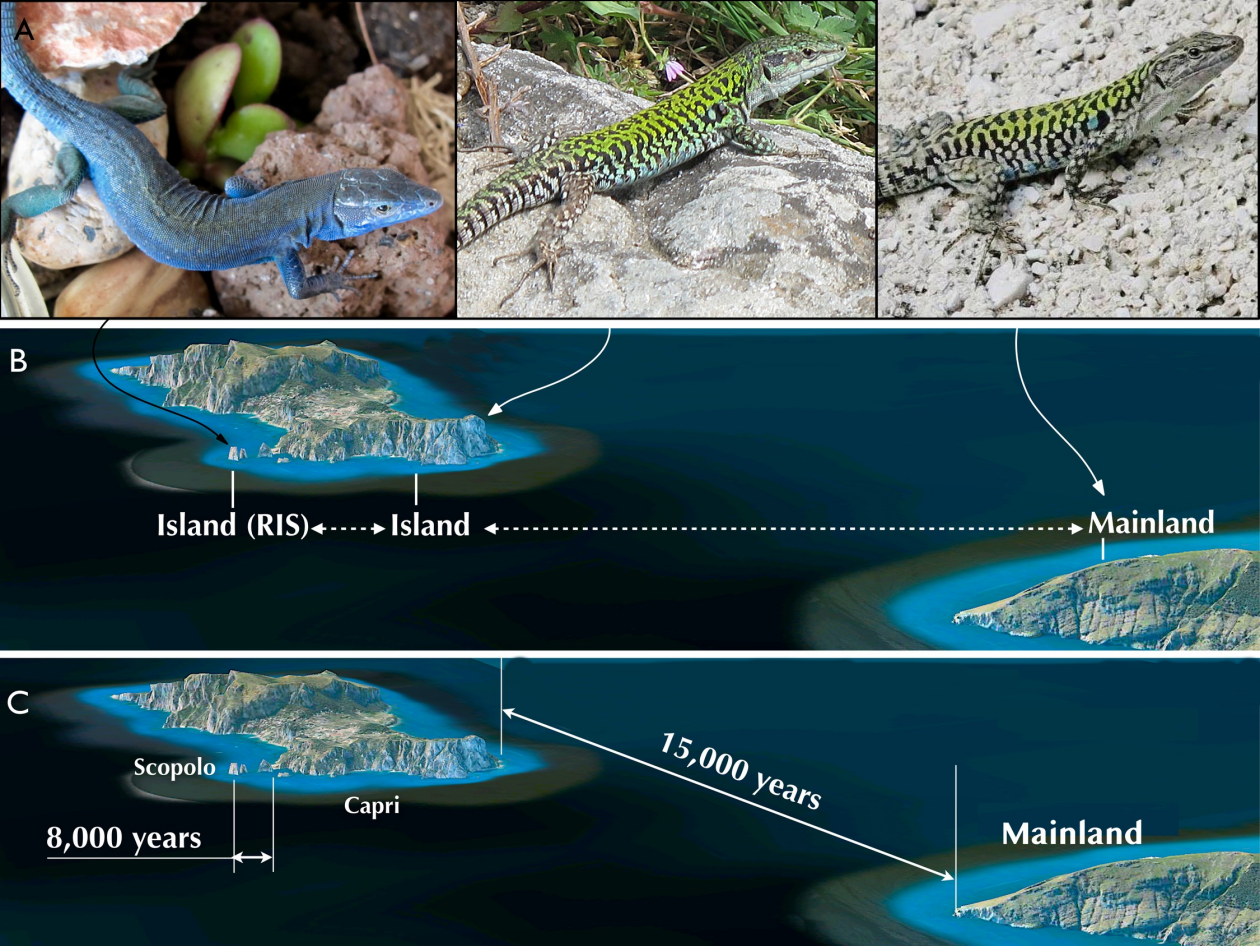
The system studied, phenotypes and population location sites. **(A)** the three typical lizard phenotypes found on Scopolo, Capri and the mainland (from left to right); **(B)** spatial location and size of the Capri two islands (Capri and Scopolo islet) off the mainland; **(C)** geological dating of the origin of the two islands. RIS indicates the Reversed Island Syndrome that we described in previous work [23–25].

Here, we analyzed a system composed of three lizard populations (*Podarcis siculus*): one of these living on the mainland and two on islands. The island closest to the mainland is a big island called Capri (in Gulf of Naples, South Italy) meanwhile the second islet is Scopolo, close to Capri and inhabited by blue lizards (Fig 1). Despite a short distance among the mainland, Capri and Scopolo, different *P*. *siculus* subspecies have been described on the islet: *P*. *siculus coerulea* (Eimer 1872) (left in Fig 1A). This latter is characterized by melanic blue colouration [22,26,27], demonstrated as a spandrel effect of the RIS [24]. The wild-type subspecies *P*. *siculus siculus*, typically characterized by a white abdomen and a green back is found on Capri and mainland (middle and right in Fig 1A).

Herein, we analysed the complete mitochondrial genome (mtGenome) to infer molecular evolution across lineages of lizard populations and sequenced brain transcriptomes to investigate differential nuclear gene expression.

The mtGenome is an excellent tool to study rates of molecular evolution, genomic structure and function, phylogenetic relationships and biodiversity [28]. Moreover, the mtDNA substitution rates in a lineage could be associated with the substitution rates in the nuclear DNA [29]. Hence, the mtGenome could be a proxy to make inference on the nucleotide substitution rate in nuclear genomes [29]. Another line of thought suggests that mtDNA has a faster mutation rate and matrilineal inheritance, and it is under different selection pressure than the nuclear genome [30,31]. In consideration of these two contrasting points of view in our paper, we limited our inferences on the mtGenome. Mitochondrial genome evolution in our three lizard populations was calibrated using island geological age to determine the speed of change of populations in our system.

Transcriptome responses to environmental changes in lizards would elucidate how they adapt to local environments. Transcriptome-wide analysis allows the evaluation of gene expression levels of thousands of known and *de novo* identified transcripts, replacing traditional single-gene approaches [32]. RNA-seq is one of the most efficient and cost-effective methods currently available for gene discovery and gene expression analysis in non-model organisms.

Under our hypothesis, the adaptations developed by Scopolo population (under RIS) were the result of changes in gene expression [22,24,25]. This hypothesis implies that after mutational events, genes underwent the DNA fixation process in the island population, as suggested by observing mtGenome, with a simultaneous transcriptome plasticity which leads to the differential gene expression, in response to selection. Thus, the mutation rate found in the population of Scopolo should be significantly slower than in isolated population, but not influenced by very strong adaptations, as under RIS (i.e., Capri). This lower mutation rate observed on the mtGenome of insular populations, such as Scopolo, could lead to an erroneous conclusion about a lower speed of the evolution. Whereas, it is necessary to take consider that the response to natural selection could result in epigenetic events rather than mutations.

## Materials and methods

### Sampling and study area

Lizards were collected during March and April 2017, using a nylon loop and only 3-year-old males were considered for subsequent analyses. Lizards were aged using their snout-vent length (SVL) which is correlated with age [24], applying standard skeletochronologic protocols, already adopted for other poikilotherms [33,34].

Skeletochronology was performed both on phalanges and femora of euthanized animals. Bones were decalcified with 3% nitric acid for a time ranging between 1h30' and 3 h depending on their size, rinsed in tap water and cross sectioned using a cryostat. Diaphyseal cross sections (12 μm thick) were stained with Ehrlich's haematoxylin (20') and mounted in aqueous resin. Periosteal lines of arrested growth (LAGs) were then counted by using a light microscope equipped with an image analyser. We assumed that each LAG corresponds to an annual arrest of individual growth. Therefore, a lizard's age in years equals the number of visible LAGs. Growth rate (in mg/day) was estimated using least squares regression of body weights of individuals versus the number of LAGs transformed into days by assuming each LAG represents a 365-day interval. Individuals with shed tails and individuals in evidently poor conditions (either badly wounded or loaded with parasites) were excluded from the analyses.

The animals were kept with the authorization of the Ministry of the Environment and Protection of Land and Sea (also known as MATTM) (prot. 4363/2015) and with the permission of the local county authority (Cilento, Vallo di Diano e Alburni National Park, prot. 2013/0010678). Experimental procedures were approved by the Ethical Committee for Animal Experiments, University of Naples Federico II (ID: 2013/0096988) and were performed according to Italian law.

The study area involved the mainland and two islands: Punta Campanella and Punta Licosa promontories represent the mainland (geographical coordinates: 40°34′N, 14°19′E and 40°15′N, 14°54′E, respectively); Capri is a big island (approximately 1.030 ha, geographical coordinates: 40°33′N, 14°13′E); and Scopolo is an islet (approximately 0.76 ha, geographical coordinates: 40°32′N, 14°15′E).

### Mitogenome analyses

A total of 15 mtGenomes (equally distributed on Punta Campanella, Capri island and Scopolo islet) were sequenced. Whole mtDNA extractions (Punta Campanella, N = 5; Capri island, N = 5; Scopolo islet, N = 5) were performed using PureLink HiPure Plasmid DNA Purification Kits (Invitrogen), according to manufacturer’s recommendations, except for using 25 mg of tissue and initial mechanical disruption of samples for 1 h in a Phosphate Buffered Saline [35] Blank extractions without samples were systematically performed to monitor potential cross-contamination.

Before sequencing, we checked the concentration and quality of the DNA extracted using the Qubit Fluorometer 3.0 (Thermo Fisher Scientific) and the Agilent 4200 TapeStation system (Agilent Technologies), respectively.

Large-scale sequencing of the total mitochondrial genome was performed on the Illumina 2500 platform (Illumina) following the Nextera XT DNA Sample Preparation protocol as reported in manufacturer’s guidelines, at Genomix4Life s.r.l. (http://www.genomix4life.com/it/).

FastQC software v0.11.4 (http://www.bioinformatics.babraham.ac.uk) was used to perform a quality control check on the short raw reads obtained from the high throughput sequencing.

Trimmomatic software v0.35 [36], in the paired-end mode, was implemented to trim and crop the low quality raw reads (Q < 28) and to clip the primers and Illumina adapters. Paired reads were processed simultaneously, and orphan reads were removed. The remaining filtered reads were retained for subsequent analysis.

For each sample, we performed a *de novo* assembly using Geneious software v. R9.1 [37] in mapping assembler mode, and the filtered reads were used to create overlapping and contiguous sequences (contigs). To check the quality of assembly, we aligned our contigs dataset to the *Podarcis siculus* complete mitogenome [accession number NC_011609].

All mtDNA sequences (17,320 bp) from this study have been deposited in GenBank (accession number MH046197 and MH157265 to MH157278).

To assess the genetic variation of lizard mitogenomes, we measured the levels of molecular diversity of mtDNA in DnaSP: number of polymorphic sites (*S*) and of haplotypes (*H*), haplotype diversity (*Hd*), and variance of haplotype diversity (*Hd* variance).

To examine phylogenetic relationships among island and mainland lizards, Bayesian analyses were performed in MrBayes 3.1.2 [38] using *Podarcis siculus* [accession number NC_011609] as the outgroup. Statistical selection of best-fit models of nucleotide substitution was conducted through JModelTest v.2.1 [39,40] for 1) the entire mtGenome alignment, 2) coding DNA sequences, and 3) neutrally evolving noncoding regions based on the corrected Bayesian information criteria (BIC). The HKY+G nucleotide substitution model was adopted for the entire mtGenome and coding DNA sequences (BIC = 598,612,317 and 396,217,051, respectively), while the TPM2uf+G model was used for noncoding regions (BIC = 183,113,437) as indicated by JModelTest. The best fit parameters were included as priors for MrBayes 3.1.2 [38], and eight Markov Chain Monte Carlo (MCMC) searches were run for 2 million generations. For each MCMC, a tree was sampled every 1,000 generations. Tracer 1.6 [41] was used to summarize Bayesian analyses, to inspect the validity of the burn-in fraction applied and to evaluate the convergence. The first 10% of samples were discarded as burn-in [41], and convergence was assessed by checking the log likelihoods, the average standard deviation of split frequencies (<0.01), and the potential scale reduction factor in MrBayes. We also assessed convergence by visual inspection of the trace and the estimate of the effective sample size (ESS>200). A consensus tree from the retained trees was computed with MrBayes.

The distribution of the tree of the entire mtGenome alignment was visualized with DensiTree v. 2.1 [42], using the same parameters described above, with burn-in of 10%. DensiTree illustrates areas where many trees support the topology and branch length as densely coloured, whereas areas where there is more uncertainty are aggregated as a web of lines. Similarly, ambiguity in node position is smearing around the mean node height. In contrast to summary trees and clade sets, DensiTree represents a qualitative approach to tree set analysis.

Estimates of divergence time between island and mainland lizards were conducted using BEAST v.1.7.5 [43] on the mtGenome alignment according to a strict clock and under the same HKY+G model.

Calibration points for molecular divergence time estimation were obtained from biogeographical events based on sea-level rises. The latter showed that Capri island was connected to the mainland during glacial maxima but remained isolated between approximately 14 and 12 ka BP; Scopolo started to emerge as a headland between approximately 10 and 8.5 ka BP [44,45].

Tests of selection based on McDonald-Kreitman [46] and Tajima’s D [47] were computed with DnaSP 4.0 [48]. The McDonald-Kreitman test, performed for each population and for all coding regions, evaluates the prediction that if both synonymous (silent, *d*_S_) and nonsynonymous (replacement, *d*_N_) mutations are neutral, then the ratio of synonymous to nonsynonymous polymorphisms within a species will be like the ratio of synonymous to nonsynonymous divergences between population (fixed differences). We also reported the neutrality index (NI) [49], which shows the directionality of the McDonald-Kreitman test. An NI value >1 is consistent with negative selection, while an NI value <1 is consistent with positive selection.

### Transcriptome analyses

To infer modulation of gene expression between Scopolo and wild type lizards from mainland, we performed transcriptional profiling of three adult male brains from each population.

To perform differential gene expression analysis, total RNA was extracted as described in Trapanese et al., 2017 [25]. Indexed Illumina libraries were prepared from 1 μg of each purified RNA sample using TruSeq Stranded mRNA Sample Prep Kits (Illumina, San Diego, CA, USA) according to the manufacturer’s instructions. Paired-end sequencing was performed on an Illumina HiSeq 2500 System at the Genomix4life s.r.l. (http://www.genomix4life.com/it/), resulting in a total of 94,136,322 raw read pairs. Transcriptome sequencing data were deposited in NCBI SRA archive with the following accession numbers: Scopolo (SRX4071992; SRX4071991; SRX4071990), Mainland (SRX1745120; SRX1745118; SRX1745113). Read quality was assessed by FastQC software v0.11.4 (http://www.bioinformatics.babraham.ac.uk), and read quality trimming was performed by Trimmomatic software [36]. A transcript catalogue was *de novo* assembled using the whole quality trimmed read data set, concatenated into two paired fastq files. The Trinity assembler [50], run with default parameters and -SS_lib_type RF, -jaccard_clip, -normalize_reads flags set, resulted in 173,183 assembled transcripts with an N50 value of 1,997. The quality of the assembled transcriptome was assessed by BUSCO v3 pipeline [51] and the level of chimeric transcripts was assessed by standalone BLASTn analysis using as reference the recently published *P*. *muralis* transcriptome dataset [52] (S1 Table). Transcript-level quantifications for each sample were done using RSEM software [53] and the Bowtie aligner [54], as implemented in the Trinity software package. Differential gene expression was analysed using edgeR software [55], with cut-off values of FDR < 0.001 and FC > 2. Differentially expressed transcript annotation and GO-enrichment analysis (P < 0.05) were performed using Annocript [56,57].

## Results

In all three populations, we identified 11 different haplotypes. Interestingly, no haplotype was shared among populations, with 3 haplotypes found exclusively on Scopolo islet and 4 haplotypes found only on Capri island.

Genetic variability analysis among mtGenomes was performed for the entirety of the populations as a whole and for each population separately. Mainland lizards had higher diversity overall than other populations (Table 1); unexpectedly, Scopolo showed a higher level of variance of haplotype diversity.

**Table 1:** Genetic diversity observed in mainland and island (Capri and Scopolo) populations (complete mtDNA, 17,320 bp).

	*N*	*N*		*S*	*H*	*Hd*	*Hd variance*	*Hd SD*	*Tajima’s D*		*pi*
**All**	15	17,320	307		11	0.962	0.00107	0.033	0.43194	> 0.05	
**Mainland**	5	17,320	78		4	0.900	0.01600	0.126	-0.37274	> 0.05	
**Capri**	5	17,320	64		4	0.900	0.02592	0.161	-0.32525	> 0.05	
**Scopolo**	5	17,320	36		3	0.800	0.02688	0.164	1.87638	< 0.05	

*N*, number of sequences; *N*, total number of sites; *S*, number of polymorphic sites; *H*, number of haplotypes; *Hd*, haplotype diversity; *Hd variance*, variance of haplotype diversity; and *Hd SD*, standard deviation of haplotype diversity; *Tajima’s D*, value of Tajima’s D test; *pi*, statistical significance of Tajima’s D test.

**Fig 2:**
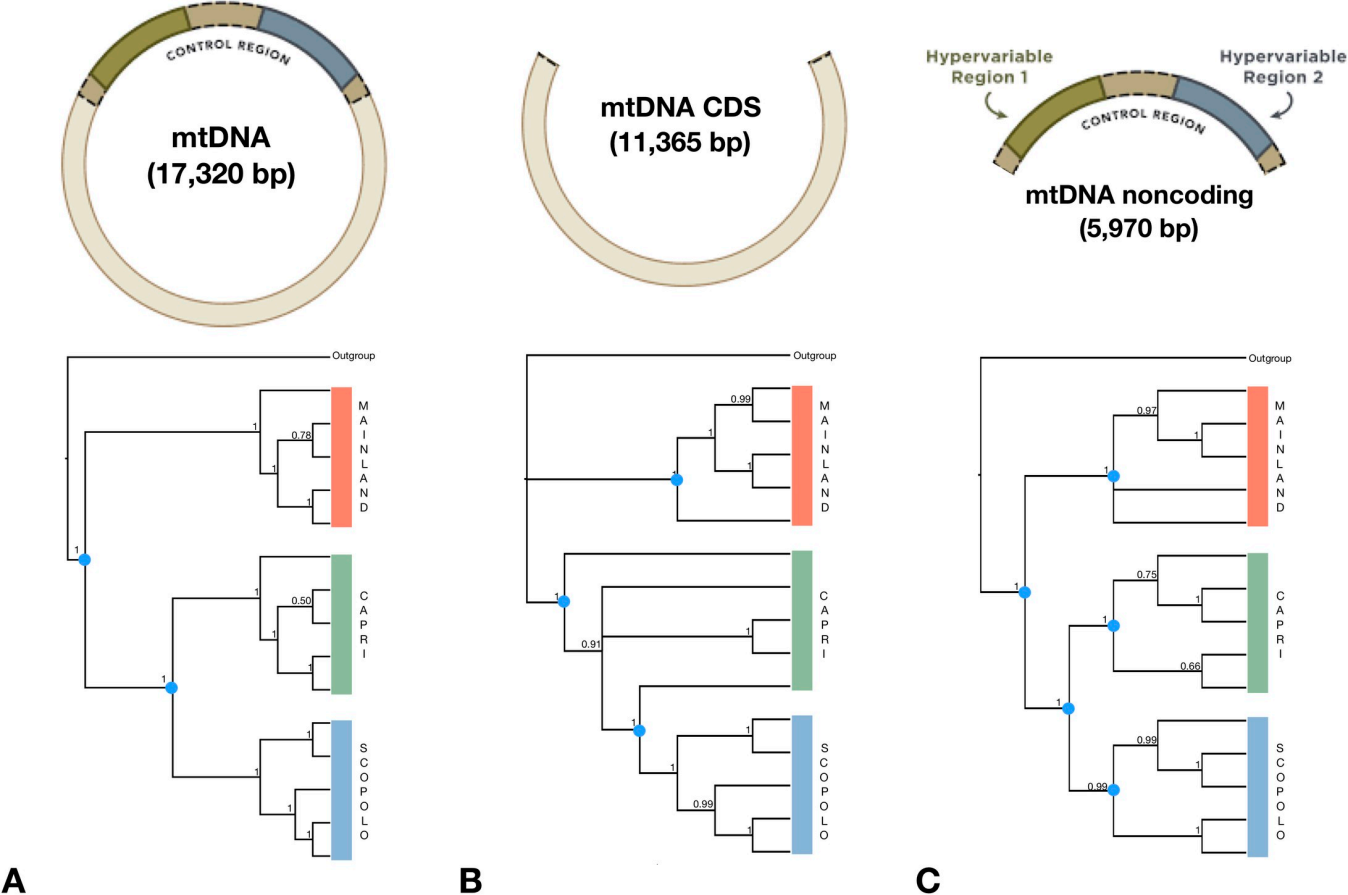
**Phylogenetic Bayesian tree of the all populations based on complete mitochondrial DNA sequences (A); coding regions (B) and noncoding regions (C).** The tree was rooted using the mtGenome of *Podarcis siculus* from Sicily (accession number NC_011609). Bayesian posterior probabilities are indicated at nodes. Colours of subclades indicate populations from mainland (orange), Capri (green) and Scopolo (azure).

**Fig 3:**
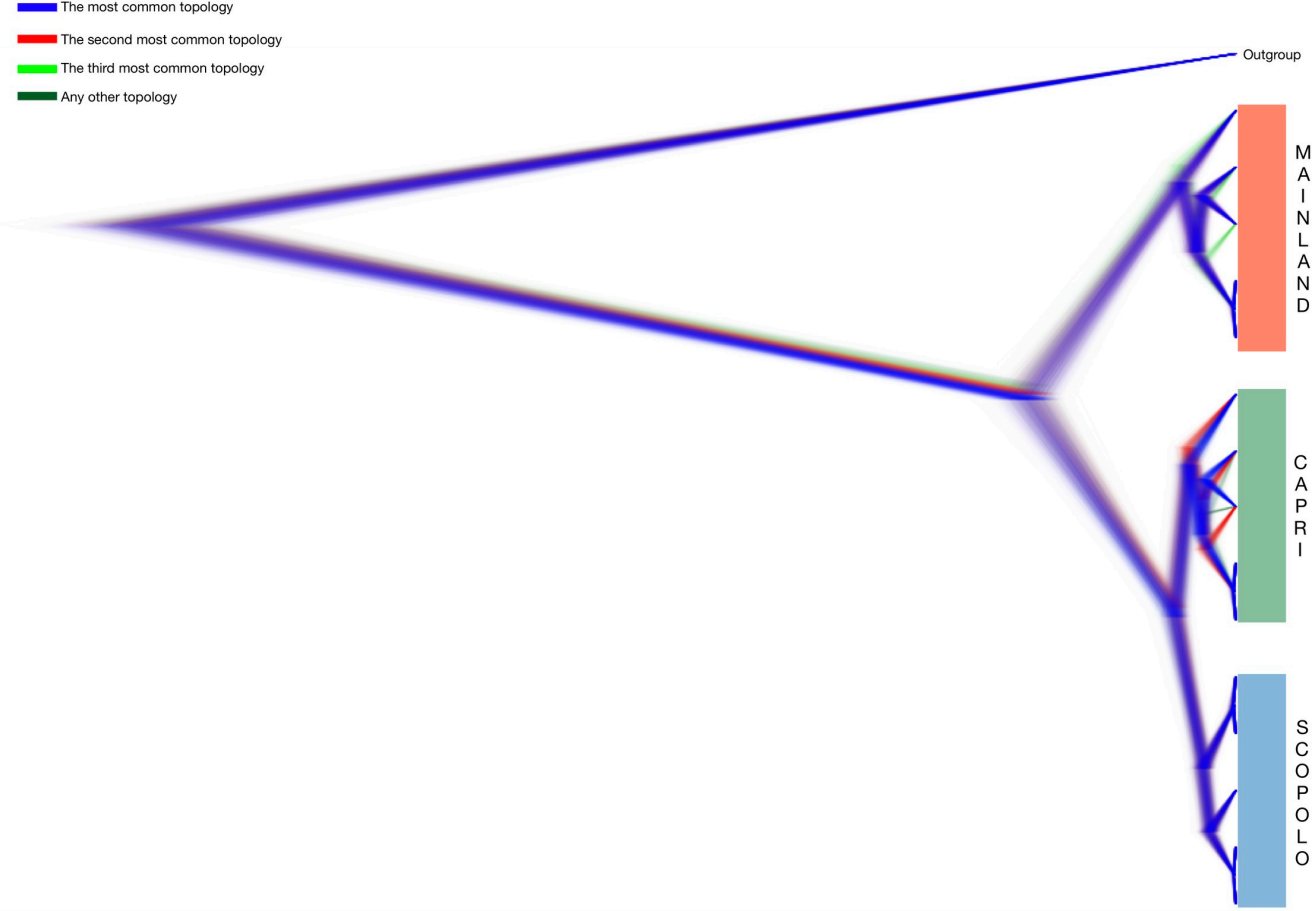
Population tree inferred with *BEAST visualized using DensiTree [40]. All trees created in the analysis (except the burn-in phase) are displayed. Trees with the most common topology are highlighted in blue, trees with the second most common topology in red, trees with the third most common topology in pale green and all other trees in dark green. On the right, colours of subclades indicate populations from mainland (orange), Capri (green) and Scopolo (azure).

Bayesian phylogenetic analysis revealed evolutionary relationships arranged in monophyletic lineages, in which the 15 mtGenomes clustered in well differentiated groups (Fig 2). The three topologies, considering the complete mitogenome (Fig 2A), the coding regions (Fig 2B), and the noncoding regions (Fig 2C), suggest that the populations of Scopolo and Capri share more recent common ancestor with each other than either it does with the mainland population. The tree set with a dominant topology in Bayesian analyses of the mtGenome sequences produced the distribution and origination time of the Most Recent Common Ancestor (tMRCA) (Fig 3). It confirmed the position of both the mainland, Capri and Scopolo origins and all the internal nodes within the radiation. All postburn-in trees are shown with their estimated branch-lengths and topologies. The fuzziness of the horizontal plane of the branches reflects the variation in branch lengths among estimated trees. This reconstruction also showed that Scopolo postburn-in trees were well supported by the topology (branches in blue), but the increase in uncertainty in tMRCA only occurred in mainland and Capri trees that supported an alternative topology (shown in red and green).

**Fig 4:**
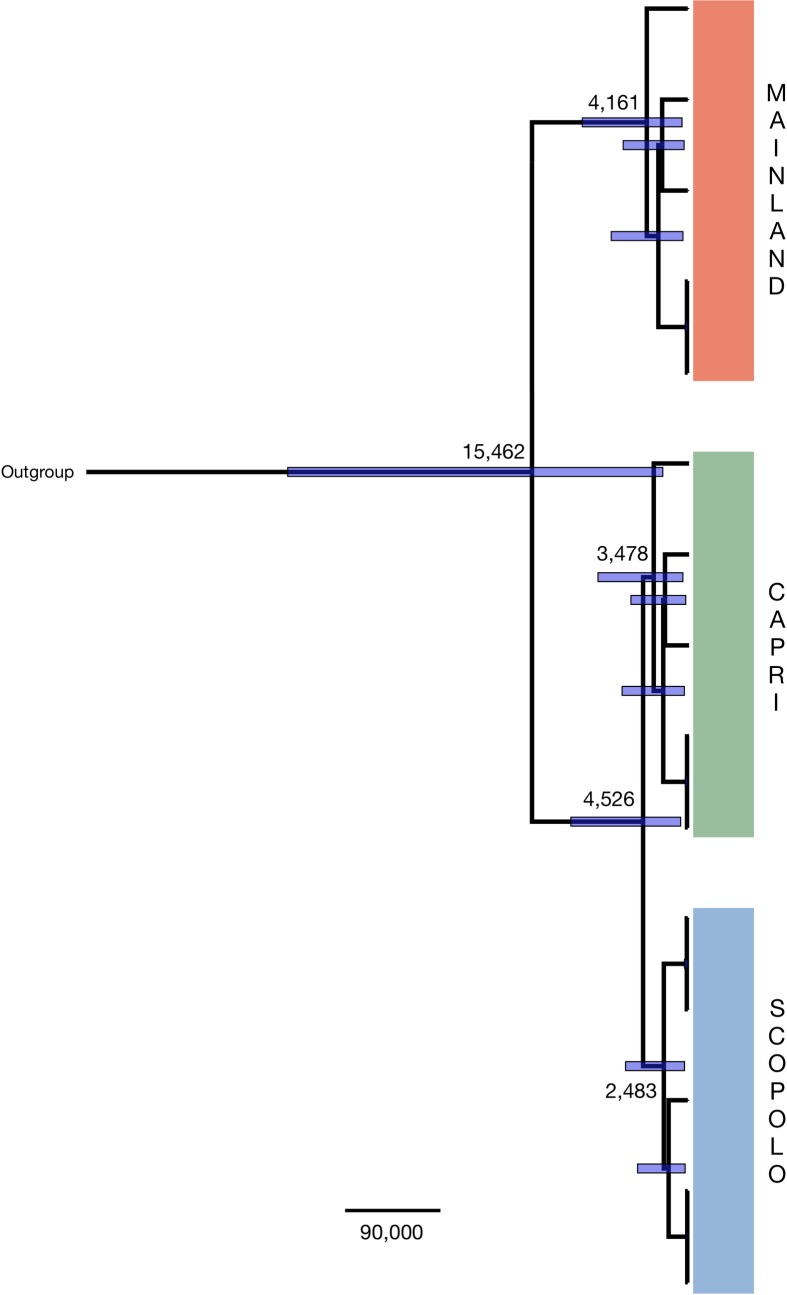
Maximum clade probability tree displayed as a chronogram from the BEAST analysis of the mtGenome alignment. All lineages evolved according to a strict clock and the HKY+G substitution model. Numbers above the nodes indicate phylogenetic support measures. Node bars illustrate the width of the 95% highest posterior density. Numbers indicate the posterior mean estimates of divergence times. On the right, colours of subclades indicate populations from mainland (orange), Capri (green) and Scopolo (azure).

The divergence time based on Bayesian phylogenetic analysis, using the Capri geological isolation date (approximately 15,000 years BP) as a prior estimate for the coalescence of all lineages, was performed in BEAST v.2.0 (Fig 4). Our results suggested that the Scopolo population diverged from Capri approximately 4,000 years BP, less than the divergence time calculated by geological observation estimated as between approximately 10,000 and 8,500 years BP [42, 43].

Due to the wide uniform priors, we used for the calibration points, the confidence interval remains large, depending on the type of analysis. However, the initial divergence within the ancestral population, from which Capri and Scopolo arose, was estimated to have occurred between approximately 3,000–6,000 years BP (range of posterior means across analyses).

Tests of selection on the mtGenome would greatly improve our understanding of how changes in gene polymorphism have been driven by selection.

A McDonald-Kreitman test on all loci combined revealed a significant departure from homogeneity comparing Capri and Scopolo populations, with a significant NI value > 1 consistent with purifying selection (Table 2). The main consequence of purifying selection is a local reduction of diversity at all levels.

**Table 2:** McDonald-Kreitman test.

	*Ni*	*α*	*G-value*	*p-value*	*Synonymous*		*Non–synonymous*			
					*Fixed*	*Polymorphic*	*Fixed*	*Polymorphic*	
**Mainland *vs* Capri**	0.773	0.227	0.711	0.399	34	44	48		48
**Mainland *vs* Scopolo**	0.650	0.350	1.944	0.163	40	41	54		36
**Capri *vs* Scopolo**	4.857	-3.857	3.905	0.048*	6	21	2		34

*Ni*, neutrality index that indicates the extent to which the levels of amino acid polymorphism depart from the expected in the neutral model [49]; Alpha value (***α***) that indicates the proportion of amino acid substitutions driven by positive selection [58]; *p-value*, probability determined by a G-test

* indicates significance at p <0.05. A particular polymorphism (synonymous or non-synonymous) is classified as being *Fixed* between species or *Polymorphic* if it is variable within either or both populations.

In other words, phenotypic variation of insular lizard is allowed only if it does not affect survivability. Accordingly, the Tajima’s D test had a significant positive value only for the Scopolo population (D = 1.876, p<0.05), suggesting the action of balancing selection with alleles maintained at intermediate frequencies.

**Fig 5:**
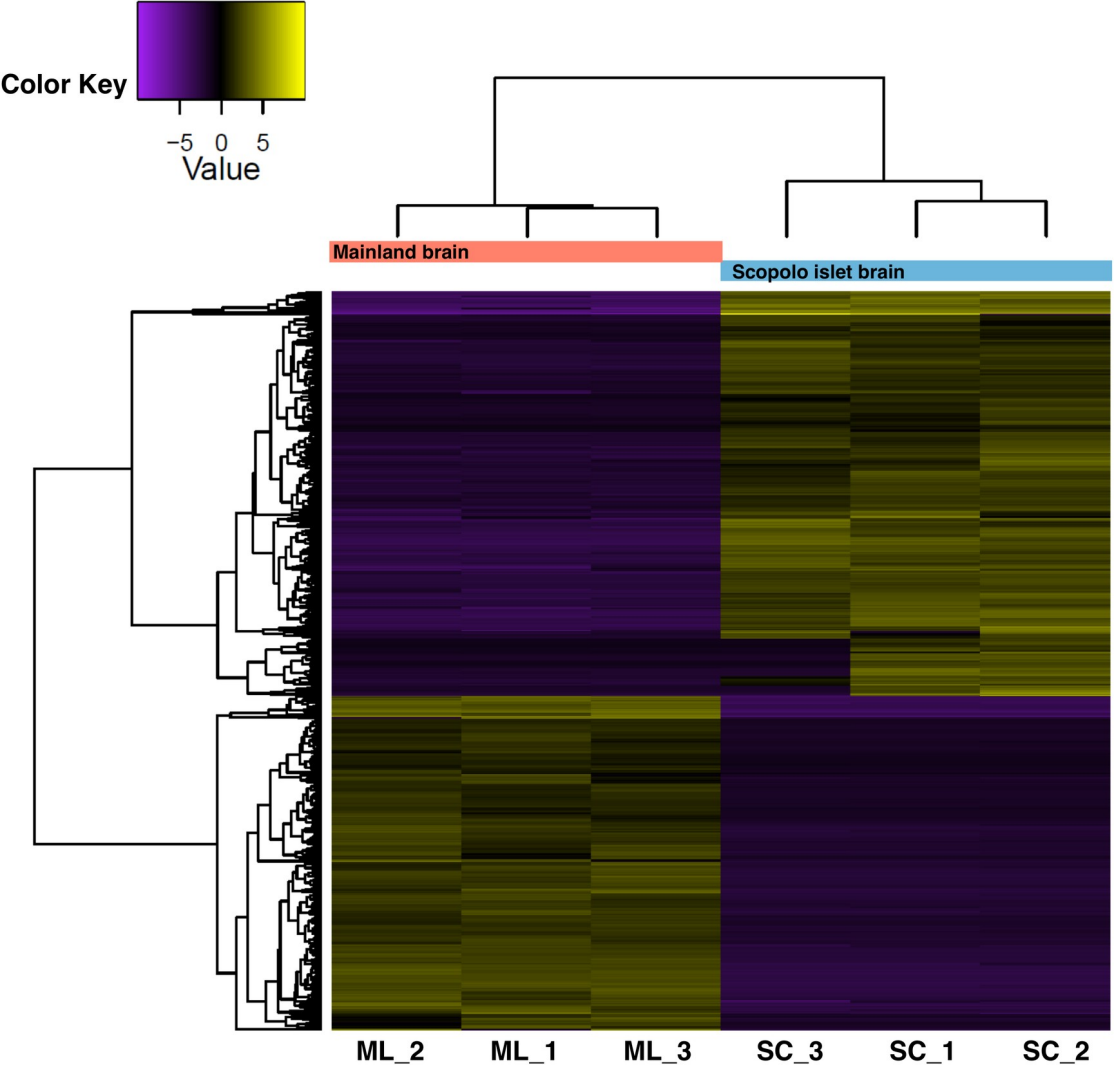
Heatmap of differential gene expression between mainland lizards and island lizards. Heatmap shows the expression of transcripts that were differentially expressed in mainland lizard brains (ML) and island lizard brains affected by RIS (SC). The heatmap gives a graphical representation of the expression level of the up and down regulated transcripts between Scopolo (SC) and mainland (ML) populations due to two the different environments. Each row corresponds to a transcript, and each column corresponds to a sample. Hierarchical clustering of differentially expressed transcripts (FDR < 0.001; FC>2) are shown from adult males of mainland (orange cluster) and Scopolo (azure cluster) specimens. Violet cells correspond to lower than average expression, and yellow cells correspond to higher than average expression.

We performed a differential gene expression analysis on brain tissues by RNA-seq to gain more information about lizards undergoing the RIS. We identified 603 upregulated and 499 downregulated transcripts in Scopolo specimens with respect to the mainland (Punta Licosa). In Fig 5, the hierarchical clustering of the differentially expressed transcripts shows two different groups corresponding to the two populations (mainland, orange cluster and Scopolo, azure cluster). Within each group, a colour gradient clearly distinguished the expression profile of the 1002 genes (upregulated in yellow, downregulated in violet), underlying the differences of the brain expression profile between Scopolo and wild type reference specimens from the mainland (Fig 5). The annotation and gene ontology analysis (GO) of the upregulated and downregulated transcripts of Scopolo island versus mainland population showed no significant GO-term enrichment. Most of the differentially expressed annotated transcripts encode for proteins involved in transcriptional and post transcriptional regulation, metabolism and neuronal development (S2 and S3 Tables). In particular, we found differentially expressed transcripts belonging to the following categories: DNA-dependent regulation of transcription, DNA repair, protein transport and translation (biological process); RNA-binding, ATP-binding and metal ion binding (molecular function); nucleus, membrane and cytoplasm (cellular component). In addition, other differentially expressed transcripts belong to the following categories: neuron projection development, neuromuscular junction development, neurotransmitter secretion (S3 Table).

## Discussion

Individual organisms can mitigate the effects of environmental variations in two ways to weaken the selection pressure in a short time: they can move to a new site according to the habitat tracking hypothesis [59] or modulate the activity of some genes to trigger phenotypic changes. Habitat tracking represents a major explanation for morphological stasis, which is one of the pillars of the theory of punctuated equilibrium [60,61]. The core idea of habitat tracking is that species remain static by actively seeking the same ecological conditions, therefore requiring little adaptive change in the face of changing environments.

On the contrary, in response to environmental factors, the regulation of the expression in some genes triggers specific adaptations, implying epigenetic mechanisms, which have been proposed as key in the plasticity model [62,63]. Some epigenetic marks, such as DNA methylation and histone modifications, can induce permanent modifications in gene expression, shaping phenotype of an organism.

Lizard populations on small islands undergoing the reverse island syndrome are ideal subjects for this evolutionary topic because rapid phenotypic changes are often visible in a short time [24,64]. These populations are constantly exposed to a wide range of environmental changes during their lifetime and across generations, in a place they are restricted to. In fact, lizards on islet (in our case Scopolo) could not adopt a habitat tracking strategy given the insular condition within strong geographic limits. As previous described, the response in terms of mutation on a specific nuclear gene may not reflect the observed phenotypic change. In fact, the changes in skin colour of *Podarcis siculus* are not associated with mutations in the coding region of the *MC1R* gene but seem to be related to a concomitant variation of expression for this gene in skin tissue [22].

We inferred the split time of the populations using geological dating [44,45], assuming this event as the origin point of new island populations. These reconstructions, compared to molecular pattern of diversification, based on mtDNA mutation rate in lizards [29], can be used to evaluate the effect of selection on the mutation rates of the populations.

Splitting events and length of branches have compared considering the geological scenario.

Mitochondrial DNA topology confirms the geological data of land separation. In fact, a common ancestor between Scopolo and Capri is more recent than what unites Capri to Mainland.

The estimation time of the coalescent lineage using mtGenomes suggests a lower rate of sequence divergence for Capri–Scopolo (4,000 years), calibrating the geological time clock on Capri–Punta Campanella that split 15,000 years BP, despite the extraordinary phenotypic variation observed in Scopolo lizards suffering RIS, characterized by strong melanization [22,65].

All the above could make it possible to interpret the observed genetic variation of Scopolo lizards in the context of adaptive plasticity. The differential expression of genes underlying some traits could lead to plasticity, adapting the same genotype to a wide range of environmental conditions. This plasticity could affect the dispersion of phenotypes under different selective pressures, promoting the genome evolution.

If lizards change based on their plasticity, the severity of the selection on these phenotypes will also change; consequently, variation on mtDNA resulting from selection may arise more rarely/slowly than expected, as we found for the Scopolo population. Interestingly, a lack of nucleotide variation on mtDNA was observed in this population, which is suffering from purifying and balancing selection, as revealed by tests. The purifying selection shaped the mtDNA evolution in animal populations, because all mtDNA genes play crucial roles in the animal’s life [31,66–68].

Indeed, Tajima’s D test and McDonald-Kreitman test for Scopolo, agree with low number of mutations at the mtGenome level compared to the expected, even if we consider the effect of population subdivision and decline (Table 2).

The hypothesis of adaptive plasticity suggests that this is the earliest key step during adaptive evolution process, driving the persistence of the populations in new environments, since that selection fixes variations [62,64,69–72].

We hypothesize that Scopolo lizards respond quickly to the environment by modulating the expression level of many genes, as highlighted by the differential gene expression analysis performed between Scopolo and mainland brain tissues. In fact, the two groups of lizards show a clear population-specific global expression pattern (Fig 5), in particular, for transcripts involved in metabolism, neuronal differentiation and transcriptional and post-transcriptional regulation of gene expression (S2 Table and S3 Table). These results support our hypothesis because metabolism represents one of the main traits involved in RIS. The lizards of these islets, where the environment suddenly changes, must make the best use of resources when available, through metabolic adaptations as well as a neuronal development that supports lizard towards stressors present on the islets. These are environments at the mercy of storm surges, predators and drought. Furthermore, it is interesting to note that transcriptional and post-transcriptional regulation of gene expression could be an indication of the ways by which lizards become rapidly adapted developing the suite of traits described in the RIS. More broadly, we are convinced that RIS could be the result of adaptive plasticity, in consideration of the times at which they arise. For example, a rapid morphological evolution has been shown in dwarf California channel islands foxes [3], many birds (especially rails) that became flightless [73], dull-coloured [74], large-billed [12,75], and larger and less aggressive rodents [76].

However, our hypothesis of adaptive plasticity will require future explicit tests to clarify the specific environmental conditions under which the syndrome may (or may not) have evolved and the molecular mechanisms underlying plasticity.

Recent studies suggest that phenotypic plasticity can be mediated through epigenetic effects [77–80], such as DNA methylation that increases variance in response to stressful conditions [63,81–83]. In the near future, it would be interesting to investigate in detail differentially expressed genes in this population. Currently, data suggest that mtDNA of the populations under RIS changes according to purifying selection. Since mtGenome responds to such selection, as a consequence the genome carries out a modulation of gene expression rather than mutation, enhancing the key role of plasticity in these unpredictable environments.

## Supporting information

S1 Table BLASTn output of 100 randomly selected DE transcripts of *P*. *siculus* against the *P*. *muralis* transcriptome database and BUSCO v3 pipeline output. (XLSX)

S2 Table Annotation table of the top 20 up and down regulated transcripts expressed in the brain tissue of the Scopolo population. (XLSX)

S3 Table Gene ontology results of the up and down regulated transcripts expressed in the brain tissue of the Scopolo population. (XLSX)
