# Correction: Fixation of genetic variation and optimization of gene expression: The speed of evolution in isolated lizard populations undergoing Reverse Island Syndrome

**DOI:** 10.1371/journal.pone.0256943

**Published:** 2021-08-26

**Authors:** Maria Buglione, Simona Petrelli, Valeria Maselli, Martina Trapanese, Marco Salvemini, Serena Aceto, Anna Di Cosmo, Domenico Fulgione

The second paragraph of the Methods section detailing methodology for skeletochronology was included by mistake; the authors clarify that skeletochronology to infer age by related snout-vent length (SVL) was not used in this study. Specimen age was deduced using SVL measurement only.

The erroneously included description of the skeletochronology is adapted from reference [24], which is cited in support of the snout-vent length methodology used to select adult lizards [1].

Accurate ageing beyond selection of adults is not essential for the study design in [1].

For mitogenome analyses, tissue samples from the terminal end of the tail were collected from live specimens. To minimize demographic impact, transcriptome analyses of brain tissue were carried out only on specimens that accidentally died during capture or manipulation (3 from Scopolo, 3 from the mainland population).

Specific sanitary protocols were employed to guarantee isolation of individuals of one population from the others to avoid the potential spread of pathologies and parasitosis. The research program for capture, manipulation, tissue sampling, temporary housing, and release of individuals at the site of capture was assessed by the Istituto Superiore per la Protezione e la Ricerca Ambientale (ISPRA) and by the Societas Herpetologica Italica and subsequently approved by the Ministry of the Environment and Protection of Land and Sea.
